# Evidence-Based Practice: Implementing REIS Findings in South African Aged Care Facilities

**DOI:** 10.1177/15394492231164948

**Published:** 2023-04-25

**Authors:** Melissa Kilian, Tania Rauch Van der Merwe, Sanetta H. J. du Toit

**Affiliations:** 1 University of the Free State, Bloemfontein, South Africa; 2 WITS University, Johannesburg, South Africa; 3 The University of Sydney, New South Wales, Australia

**Keywords:** collaborative residential aged care, nominal group techniques, occupation-based assessment tools, occupational therapy, Residential Environment Impact Scale (REIS)

## Abstract

**Background:**

Older adults living in Residential Aged Care Facilities (RACFs) in South Africa are often exposed to environments that fail to provide adequate occupational opportunities. Practitioners in developing economies are challenged to provide therapeutic services in environments that emphasize occupational deprivation.

**Objectives:**

This study investigated barriers and enablers to implementing recommendations derived from the findings generated by the Residential Environment Impact Scale (REIS).

**Method:**

A two-phased embedded mixed methodology was employed. Phase 1 was the administration of the REIS. In Phase 2, leadership staff considered factors to implementing findings of the REIS, using the nominal discussion group technique. Data were thematically analyzed.

**Findings:**

Findings indicated an organizational culture characterized by top-down management and a medically dominated care approach, constrained by financial and operational concerns.

**Conclusion:**

Using the REIS to holistically assess, RACFs are an applicable tool that could promote collaborative approaches to enhance organizational culture change, cultivating more occupational opportunities.

## Introduction

Social and economic vulnerability within the Global South (United Nations Conference on Trade and Development, 2018) continues to negatively impact the long-term aged care sector. In South Africa (SA), enabling environments within Residential Aged Care Facilities (RACFs) are limited and poorly regulated. Many state-funded RACFs do not comply with the South African Older Person’s Act 13 of 2006, an initiative by the Department of Social Development intended to uphold protection and security and promote well-being of older people. Managerial boards of RACFs complain about lacking funds or failure to receive approved government subsidies (Benade, 2012; De Jager et al., 2015; Mapira et al., 2019).

In SA, older adults, in institutional care settings, are generally viewed as well cared for if their basic needs for security, food, and personal hygiene are met. Residents living with dementia are particularly incapacitated by the daily operations in RACFs as they lack the capacity to retain a level of autonomy in the structured and medically focused routines prescribed by operational policies of facilities (Morgan-Brown et al., 2019). The best international practice in dementia care is considered to be person-centered care (PCC), a philosophy that acknowledges the individual and their preferences and needs (Fazio et al., 2018; Grabowski et al., 2014) despite their degenerative neurocognitive disease affecting cognitive confunctions such as memory, orientation, thinking, reasoning, and behavior. However, accreditation of South African RACFs is not contingent upon adhering to PCC practices and no PCC policies exist in SA. Occupational therapists, as *environmental assessors*, are skilled in creating awareness of environmental influences on PCC practice (Brodrick & Barry, 2016). Unfortunately, occupational therapists are typically not employed in South African RACFs, predominantly due to economic constraints. Staff training on dementia and PCC is limited, which impacts staff’s interactions with residents. As a result, institutionalized older adults become alienated and deprived of the opportunities to exercise control within their daily lives (Causey-Upton, 2015; Du Toit et al., 2019).

The occupational therapy profession has seen an international shift from individual intervention to a collective approach in community settings (Du Toit et al., 2019; Frank Barney & Perkinson, 2016). Collective intervention has become imperative due to the limited number of practicing occupational therapists and the financial constraints hindering older adults in low socioeconomic conditions from otherwise accessing their services. Due to scant empirical occupation-focused evidence in South African RACFs, it remains a challenge within the constrained economic context to convince stakeholders in aged care of the benefit of a collective approach to PCC.

For occupational therapists to propose environmental adaptations and introduce PCC, they need to show evidence of how residents and staff experience day-to-day routines in their RACFs and whether these routines contribute to enabling, or disabling, occupational engagement (Briller et al., 2016; Du Toit et al., 2019). Measuring engagement and occupational participation in RACFs is an effective way to do this. The Residential Environment Impact Scale (REIS) is a tool that measures the impact of the physical, social, and organizational environments on the quality of life of residents living in shared spaces (Fisher et al., 2014) and has been found to have clinical utility in Sweden (Svensson et al., 2018) and Australia (Du Toit et al., 2021). In this study, the REIS was administered in three RACFs affiliated with a nongovernmental organization in SA, serving vulnerable and marginalized populations. such as institutionalized older adults and people living with dementia (Engo, 2020). Findings and recommendations obtained from the REIS were presented to leadership staff to determine what they deem potential factors to consider implementing the tool and its findings in the respective facilities. The research question for this study was the following: *What are the barriers and enablers to implementing recommendations from the REIS within the involved organization’s RACFs?*

## Method

This study was approved by the Research Ethics Committee of the Faculty of Health Sciences at the University of the Free State (Ethics number: UFS-HSD2018/0605/1906). A pragmatic mixed-methods approach with a qualitative focus was employed and data were collected in two phases. Phase 1 commenced with the researcher executing the REIS assessment in three participating RACFs. The collated REIS findings (Table 4) provide quantitative scores based on the qualitative data collected via interviews and observations. Refer to Table 1 for a comparison of the three participating facilities. Phase 1 participants included in the interview component of the REIS were recruited via opportunistic sampling during site visits. Residents who had severe cognitive disabilities hindering their ability to understand questions during the interview were excluded. Phase 2 was a discussion group with leadership staff, using the nominal group technique (NGT). NGT participants were recruited via purposive sampling to include permanently employed leadership staff. Informed and process consent was obtained from all residents and staff participants during both phases.

**Table 1.** Comparison of the Residential Aged Care Facilities.

Feature	Facility 1	Facility 2	Facility 3
Size (m^2^)	Unknown	Unknown	8,159 m^2^
Urban/rural	Urban	Rural	Urban
Number of residents	50	16	185
Number of staff	44	24	130
Racial composition residents (*n* = number)	White – 100% (*n* = 50) Black – 0 Colored – 0	White – 87.5% (*n* = 14) Black – 12.5% (*n* = 2) Colored – 0	White – 99.4% (*n* = 184) Black – 0.54% (*n* = 1) Colored – 0
Racial composition staff	White – 25% (*n* = 11) Black – 75% (*n* = 33) Colored – 0	White – 16.6% (*n* = 4) Black – 83.3% (*n* = 20) Colored – 0	White – 14.6% (*n* = 19) Black – 83.7% (*n* = 108) Colored – 2.3% (*n* = 3)
Dedicated dementia unit	No	No	Yes
Transport	✓	x	✓
Tuck shop^a^	x	x	✓
Religious services	✓	✓	✓
Mobile food service	x	x	✓
Assisted living services	x	x	✓
Volunteers	✓	✓	✓
Occupational therapy	Intermittent	Intermittent	✓
Physiotherapy	Intermittent	Intermittent	Intermittent
Medical doctor	x	x	✓
Recreational activity program	x	x	✓
Goodwill services^b^	x	x	✓
Hairdressing	✓	✓	✓
Mobile library	x	x	✓

*Note.* ✓ = yes; x = no.

a A tuck shop is a small convenience store located on the premises of the facility selling confectioneries and essential groceries. ^b^In South Africa, goodwill services are regarded as a group of people collecting donations and distributing it to support the basic needs of people who are in need thereof.

## The REIS

The REIS was developed by occupational therapists for use in residential care settings to assess the impact of the environment by focusing on how well the environment addresses the occupational needs of residents and ultimately their quality of life (Fisher et al., 2014). The Model of Human Occupation (MOHO) is the theoretical base for the REIS that considers the environment as influencer of occupational motivation, engagement, and organization both positively and negatively. The REIS consists of four domains, namely, Everyday Space, Everyday Objects, Enabling Relationships, and Structure of Activities, and each contains five subdomains that are assessed and scored via a four-point ordinal scale. The numerical scoring is contingent on findings obtained from interviews, observation, and a walk-through of the facility, as prescribed by the REIS. The *walk-through*, observation of *three everyday activities*, and *interviews with residents and staff*, which can be done formally or informally, enable the therapist to organize observations of the environment according to the 20 items of the rating form. The interviews provide opportunities for staff and residents to comment about their living and working environment, giving the therapist further insight into the environment and how it affects daily routines. A score of 1 indicates the environment to strongly interfere with people’s sense of identity and competence by not providing opportunities, whereas a score of 4 indicates an environment that strongly supports identity and competence by providing exceptional opportunities. A detailed interview guide and report template to present findings accompanies the REIS manual. The recommendation reports provided to the participating facilities included both combined scores and scores of the specific domains and the interpretation thereof, supported by qualitative data and compiled according to the REIS template.

## Nominal Group Technique

The NGT is a structured brainstorming process that provides a systematic method for obtaining qualitative information from a group (Boddy, 2012; Harvey & Holmes, 2012). A research question is posed to the members as a group (as mentioned below in the “Procedure” section), and a systematic process is followed to first obtain individual insights from group members before systematically facilitating joint consensus as a collective on the research question initially posed. The NGT process followed the following steps: *introduction, silent generation of ideas, round robin contribution of ideas, clarification of ideas*, and private *ranking of ideas*. As a rigorous methodology, the NGT promotes equal participation for all group members, prevents domination by group members, and reduces interviewer bias (Boddy, 2012; Harvey & Holmes, 2012). The final product is a set of consensus statements in response to what the potential factors are to consider for implementing the REIS (see Table 2). The clarification and discussion step provides an opportunity for member checking, which contributes to the trustworthiness of the process and the NGT data collected.

**Table 2.** Consensus Statements Identified During the Discussion Group Process.

Ranking	Consensus statement
**1**	Staff development, needs/skills analysis, “warming the soil.” Exchange of skills
**2**	Considering residents’ specific needs. Identifying residents’ needs—guides the managers’ reaction/understanding on what a universal need is and what is a more specific need within aged care.
**3**	To focus on the relationships between care staff and residents rather than carer versus resident. Equalization can aid each other with daily tasks such as serving tea.
**4**	Optimal use of space versus homeliness: Using everything that you have or identifying how you can re-use something more effectively.
**5**	Proposed activity program with specific time, place, providing choice for participating in. Critical evaluation and reaction of the residents’ needs to ensure feasibility of activity programs.
	Effective use of existing resources and use that as a starting point to advance and improve existing features.
	Identifying gaps. REIS especially gives meaning that leads to further planning.

*Note.* REIS = Residential Environment Impact Scale.

## Field Notes

Extensive field notes, taken by the researcher, contained both in-the-moment observations and critical reflection (Phillippi & Lauderdale, 2017). During Phase 1, the researcher followed a systematic process of jotting down notes and keeping a reflexive journal. These notes were subsequently transcribed. Electronic field notes were taken during Phase 2. Both sets of notes formed part of data analysis and were thematically coded by the main author and co-coded by the co-authors.

## Procedure

Following the REIS assessments, a report containing the domain scores and supportive findings were presented to each facility. Recommendations to improve service delivery toward a PCC approach were included, such as improvements to signage, improvements to the accessibility and usability of existing environmental features, and suggestions for more consistent use of assistive devices.

Leadership staff were requested to consider the findings and recommendations prior to participating in the discussion group, so they could offer informed comments on the benefits and barriers to considering the REIS findings and implementing the suggestions.

During the discussion group in Phase 2, six management staff (see Table 3) were asked the following question: “What do you see as the potential factors to consider for implementing the REIS at your facility?” The group was facilitated by an expert NGT facilitator, and five priorities were identified during the discussion group. The first author observed the group, taking electronic field notes and co-facilitated as necessary. The group was not audio- or video-recorded.

**Table 3.** NGT Participants.

Participant	Facility	Length of facility employment (months)	Position	Gender	Race	First language
P1	1	72	Manager	Female	White	Afrikaans
P2	1	60	Registered nurse	Female	White	Afrikaans
P3	2	9	Manager	Female	White	Afrikaans
P4	2	264	Manager	Male	White	Afrikaans
P5	3	18	Nursing manager	Female	White	Afrikaans
P6	3	4	Registered nurse	Female	White	Afrikaans

*Note.* NGT = nominal group technique.

The systematic procedure of administering the REIS and the reflexive journaling, in addition to co-coding data with the co-authors, was employed as strategies to manage the researcher’s pre-understanding and personal interpretations of operational practices in RACFs and how this influences occupational participation.

## Data Analysis

The qualitative data generated during the REIS assessment and NGT (including both brainstormed ideas by each participants and nominated priority statements by the group as a whole) were analyzed using a four-step process of inductive thematic analysis, that is, *organization, perusal, classification*, and *synthesis* (Creswell & Creswell, 2017). The researcher read through the data and highlighted sections, specifically relating to the barriers and enablers of implementing the REIS (*organization and perusal*). She subsequently *classified* the data using descriptive coding (Saldaña, 2016), and finally, the data were *synthesized*. The codes were organized into categories, subcategories, and themes. Trustworthiness, considered as the “gold standard” to ensure quality (Creswell, 2014), was honored by using reflective journaling to create an audit trail during all phases of data analysis. In addition, coding and interpretation were discussed with senior co-authors. As part of the nonlinearity of qualitative data analysis, the themes that emerged from the analysis were considered in response to the research question and presented to reflect barriers and enablers within the findings.

## Results

### REIS (Phase 1)

The REIS findings indicated that each facility had similar approaches, routines, and environmental features. The REIS scores ranged between 1 and 3, and no facility scored a 4/4 in any of the categories evaluated, indicating that the collective environments of the facilities needed further deliberation and adaptations to ensure that needs of residents and staff were met. The collated numerical scores obtained from the REIS assessment at each participating facility can be seen in Table 4.

**Table 4.** Quantitative REIS Results Obtained.

		Facility 1		Facility 2		Facility 3	
Assessment Tool	Indicator		Score out of 4		Score out of 4		Score out of 4
REIS (Version 4.0)	Everyday space	Accessibility of space	3	Accessibility of space	2	Accessibility of space	3
		Adequacy of space	2	Adequacy of space	2	Adequacy of space	3
		Homelike qualities	3	Homelike qualities	3	Homelike qualities	3
		Sensory space	2	Sensory space	3	Sensory space	3
		Visual supports	2	Visual supports	2	Visual supports	2
	Everyday objects	Availability of objects	2	Availability of objects	2	Availability of objects	3
		Adequacy of objects	3	Adequacy of objects	2	Adequacy of objects	3
		Homelike qualities	2	Homelike qualities	3	Homelike qualities	3
		Physical attributes of objects	3	Physical attributes of objects	3	Physical attributes of objects	3
		Variety of objects	2	Variety of objects	1	Variety of objects	3
	Enabling relationships	Availability of people	2	Availability of people	2	Availability of people	3
		Enabling respect	2	Enabling respect	2	Enabling respect	2
		Support and facilitation	2	Support and facilitation	2	Support and facilitation	2
		Provision of information	3	Provision of information	2	Provision of information	3
		Empowerment	2	Empowerment	2	Empowerment	2
	Structure of activities	Activity demands	2	Activity demands	2	Activity demands	3
		Time demands	1	Time demands	1	Time demands	2
		Appeal of activities	1	Appeal of activities	1	Appeal of activities	3
		Routines	2	Routines	2	Routines	2
		Decision making	2	Decision making	2	Decision making	3

*Note.* REIS = Residential Environment Impact Scale.

### Discussion Group Using NGT (Phase 2)

Table 3 summarizes the identified five factors discussion group members jointly prioritized for consideration when implementing the REIS. The most important factor that needed consideration was the need for staff development. However, managers did not specify what development opportunities were needed and seemed uncertain about operational staff members’ existing skills. The discussion phase of the NGT group provided the realization that an audit was necessary to identify the current level of staff skills at each facility.

The NGT data revealed managers had not fully considered factors to implement the findings of the REIS assessment tool as presented in their report and rather used the opportunity of the group session to discuss operational challenges within their respective RACFs. Although not documented within the list of consensus statements, participants also pointed out that the REIS assessment does not consider the financial position and constraints of the facility, and therefore, Statements 4 and 5 focused on more effective use of, or re-use of, existing resources.

### Barriers and Enablers to REIS Implementation

**Figure 1.**
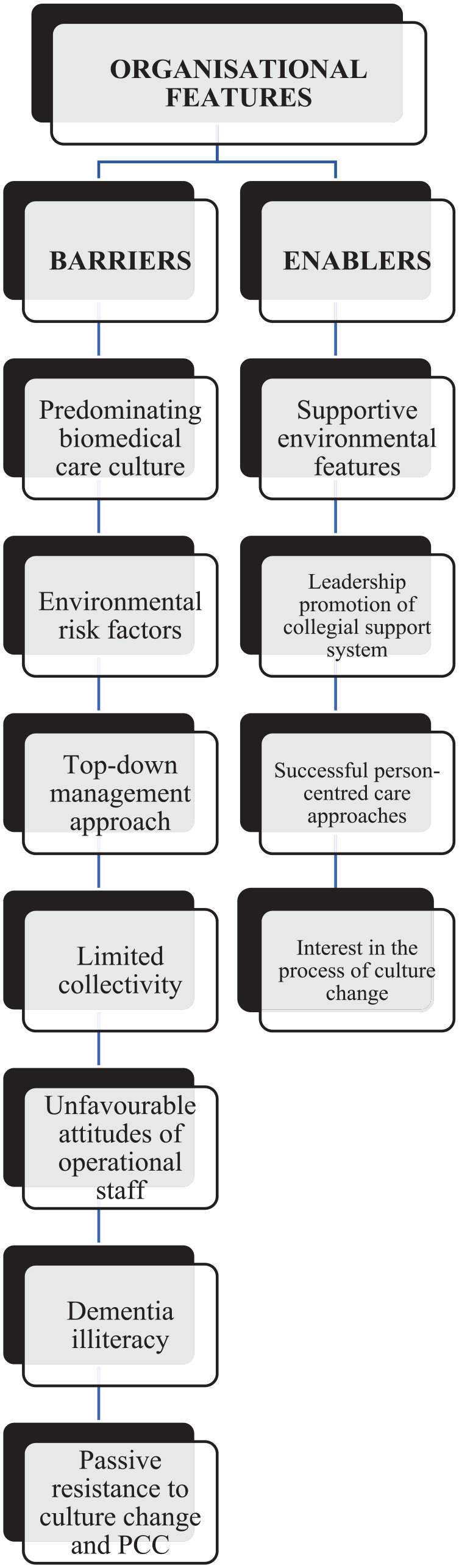
Themes Related to Organizational Culture and the Implementation of the REIS. *Note.* REIS = Residential Environment Impact Scale.

The combined thematic analysis of the data generated seven themes in relation to considerations for implementing the REIS and these are organized to highlight the barriers and enablers to the program. These themes are illustrated in Figure 1.

#### Organizational Features Presenting Barriers to Culture Change

##### Predominating Biomedical Care Culture

The imperatives of all facilities were those of a “traditional” way of doing things associated with the biomedical care culture usually present in hospitals, that is, structure, inflexible routines, and a strict focus on physical care, referring to residents as “patients.” Daily activities and routines of staff indicated a task-oriented approach and care practices focused on rules and nursing care rather than focusing on the residents’ abilities and occupational needs:

Rules, obviously, need to be very strict. (Comment, NGT participant)

Registered nurses commented on how medication management was of utmost importance and took up most of their time, preventing their spending quality time with residents.

##### Environmental Risk Factors

The environments at all three facilities included institutional trademarks such as long hospital-like corridors with centrally located nursing stations, call bells, shared rooms and cupboard spaces, large hostel-like dining rooms, large fluorescent lights throughout corridors, and at one facility, hospital beds and curtain divisions. Residents and staff did not fully utilize all the existing resources and infrastructure such as lounges, outside areas, activities presented, and assistive devices. At Facility 2, a newly renovated walk-in shower was used as a storeroom because staff became wet in the process of assisting residents with showering and refused to use it. At Facility 3, an available hoist and draw sheets to minimize impact on residents’ skin and carers’ joints was rarely used due to staff time constraints as staff indicated:

They do not have time to fetch the device and there are many residents to help. (REIS Interview-notes: Facility 1)

Environmental risk factors were associated with auditory overstimulation, uneven walk surfaces, inaccessible bathrooms, inadequate grab rails, furniture that did not support safe sit-to-stand mobilization, and flooring that presented falls-risks and was disorienting for people living with dementia.

##### Top-Down Management Approach

Managers noted they were consumed with administrative duties.

They had very little time for meaningful interaction with residents and alluded to this as a possible reason for losing touch with resident’s needs and thus a PCC approach. The facilities are managed in a hierarchical system where management is viewed as the executive body of power, decision-making, and teaching.

Power relations influenced the interaction staff had with each other, “Now I’m the big boss” (NGT Participant comment), and influenced residents. Residents seemed to fear vindication and indicated they preferred to refrain from providing specific answers during the REIS interviews:

I’d rather not say anything,” and “don’t say it was me who said that. (REIS Interview-notes: Facility 2)

Care staff voiced dissatisfaction with their salaries and working conditions and seemed preoccupied with their own needs. The current traditional care culture was viewed as maintaining the status quo and not a problem or something that needed changing or improving.

##### Limited Collectivity Between Stakeholders

Limited community and collaboration at the facilities was illustrated by (a) the disengagement between staff (operational and senior staff) and residents, and (b) the disengagement between staff and volunteers.

Disengagement between staff and residents was evident in the lack of collective participation in meaningful tasks other than personal care. Transfers, a basic mobility activity of daily living, were done *for*, or *to*, residents rather than *with* them. Staff occasionally brought residents to activities but did not regard this as an interactive opportunity. Residents, however, were observed to seek out interaction with staff by means of initiating jokes and showing more alertness when staff entered a room. One example where the staff members’ presence elevated the levels of engagement of residents was when she sang to them while serving tea and coffee.

Operational staff seemed comfortable with mostly providing care or serving residents and equally residents were accustomed to receiving such care.

Staff focused on residents’ inabilities, rather than abilities, and stated that they do everything for the residents. Time constraints were one of the main reasons staff provided for taking over care tasks rather than providing opportunity for co-occupation with residents. Managers were aware of inconsistencies in staff’s level of operational skills and associated discrepancies in the level of care staff provided: “They can learn from each other, not necessarily only from a senior staff member” (NGT Participant comment).

There was also disengagement between staff and volunteers. Volunteers felt that staff deliberately ignored them as they anticipated the volunteers would expect them to help and thus increase their workload with residents in any leisure activities facilitated by volunteers, rather viewing it as time to rest and retreat to the staff room.

##### Unfavorable Attitudes of Operational Staff

At all three facilities, when the “job was done,” care staff sat around socializing with each other, using their phones, or supervising, rather than interacting with, residents.

Service rosters and work shifts were vexatious to care staff as they experienced a heavy workload and disliked being shifted around to various departments. At Facility 3, some operational staff and residents commented on the highly strung attitude of staff in various departments. Observations at Facility 3 noted that reception staff had to deal with the issues of residents, staff members, family members, and external service providers. There was little respite for these staff as there was a constant influx of people and situations that demanded their attention.

##### Dementia Illiteracy

Residents without dementia found the behavior of residents with dementia irritating, and sometimes frightening, and expected them to be moved to a separate dementia-allocated wing. Care staff’s inability to adequately respond to some residents was observed. Staff regarded residents with dementia as “difficult” to handle and expected these residents to be confined to an isolated unit as they increased their workload.

##### Passive Resistance Toward Culture Change and PCC

Staff displayed passive resistance toward culture change, indicated by hesitancy, uncertainty, and ambivalence during the study. Some managers displayed resistance toward participating in the research process evident by comments made, periods of silence, and body language during the NGT. Managers also distanced themselves from responsibility for facilitating culture change: “I am glad I am not the occupational therapist because she has her work cut out for her” (NGT Participant comment).

Comments from managers indicated familiarity with culture change terminology; however, the concept of PCC implementation toward culture change was not properly understood. Many comments from managers and operational staff indicated that residents are viewed as a business commodity. The most frequently stated reason as to why PCC and culture change was unattainable was that the facilities needed additional finances to approach it. Managers stated that the REIS does not account for a facility’s financial position and noted that as a weakness of the assessment.

Residents’ institutionalization was offered as another reason for PCC not being implemented as “residents prefer things the way it has always been” (NGT Participant comment). Staff comments also suggested that they strongly associate PCC with recreation activities and an activity program at the facility: “One should evaluate why it (participation in activities) decreases because we want it to be feasible for the residents—person-centred” (NGT Participant comment).

#### Organizational Features Enabling Culture Change

Four subcategories illustrated the organizational features enabling culture change and the potential to implement the REIS (see Figure 1).

*Supportive environmental features* were evident at all three facilities. These included communal rooms that were sunny and inviting, smaller spaces that promoted engagement with people and the environment, culturally appropriate décor that facilitated reminiscence, and personalized cues that promoted orientation and belonging. Other features included assistive devices and structural features of the environment that contributed to the safety and well-being of residents.

##### Leadership Promotion of Collegial Support System

There were examples of management positively considering staff needs. Staff at Facility 3 mentioned the pleasure they experienced from a new staff cafeteria, which was a beautifully inviting space with couches, a kitchenette, and a TV.

Facility 3 also provided office staff with a mid-afternoon to themselves once a month, which they appreciated. Facility 3 funded a wellness program for staff that included paid-for visits to a general practitioner, assistance with health devices such as glasses and hearing aids, access to medication for lower-income staff members, and weekly exercise classes.

##### Successful PCC Approaches

Despite the predominant biomedical model toward nursing care, there were examples of successful PCC approaches indicating consideration of individual needs:

We must understand that it is their home, we work in their home. (NGT Participant comment)

It’s absolutely true, every person is unique . . . and if I know that then I can understand the resident much better. (NGT Participant comment)

##### Interest in the Process of Culture Change

Despite limited understanding of the core concepts of PCC, RACFs aspired to promote the dignity of residents. For example, at one facility, staff presented leisure activities on an individualized basis, breaking down the activities step-by-step to promote better engagement. Overall, management experienced the NGT discussion as a meaningful opportunity to discuss similar challenges they experienced. Participants requested the NGT process be duplicated with the rest of the management team at each facility:

## Discussion

The REIS assessment findings indicated there was much room for environmental improvement in each facility. However, findings also indicate that there are care culture issues that need to be addressed before any REIS assessment can be actioned within a facility.

These care culture issues are primarily to do with the organization’s operational culture that is geared toward a top-down management approach and biomedical care practices of operational staff that result in occupational injustices that are not fully considered by management. The findings suggest that management perceive PCC primarily as an additional financial burden and operational staff perceive it in terms of additional workload.

Staff had limited understanding of the concept of culture change and PCC. This finding is congruent with that of Du Toit and Buchanan (2018) whose international investigation of management perceptions of PCC (which included South African managers) found that although managers of residential facilities might claim to “do” PCC, their understanding of the approach is limited.

It is the responsibility of management to guide operational staff through the culture change process (Du Toit et al., 2020; Thomas et al., 2014). Operational staff have a constant and direct impact on the lives of residents and are therefore key stakeholders in successful culture change. This study suggests a cultural gap for staff regarding caring for residents, versus ensuring that their own needs are fulfilled. Operational staff’s primary concerns were based on being understaffed, underpaid, experiencing racial marginalization, and frustration dealing with residents with dementia. These concerns have also been noted previously (e.g., Du Toit & Surr, 2011). The high unemployment rate (35.5% for females and 32.6% for males; Statistics SA, 2022) and the perceived low skill level needed to perform care work have established an aged care workforce without the necessary training. Many are desperate for any work and employers are able to maintain financial viability by paying their employees minimum wage (South African Human Rights Commission, 2015).

There has been a growing critique of PCC as unattainable within the limited resource constraints of most RACFs (Harding et al., 2015), which this study corroborates. A lack of human and financial resources created a regimental focus on maintaining order and organization, placing tasks before people. Management pointed out that the REIS assessment does not consider the financial constraints of the facility. A similar implementation issue was raised in the Australian RACFs context (Du Toit et al., 2021).

It cannot be ignored that the three participating RACFs needed to remain financially viable within the constrained economy of SA, and perhaps even more so with the compounded global economic pressures resulting from the COVID-19 pandemic. It is very likely that any financial support to state-subsidized RACFs will be reduced in the future.

The organizational care culture of the organization in this study is exacerbated by poorly regulated national oversight. Internationally, but even more so in SA, low priority is attached to investigating and promoting health and well-being policies for older adults, particularly long-term institutionalized older adults (Benade, 2012; De Jager et al., 2015). While there is lax oversight by national governing bodies, incentive for RACFs in SA to use these tools is arguably reduced.

However, the opportunity, presented by this study, to discuss the findings of the REIS and reflect upon it, did provide management with valuable insights. They noted their experience of participating in the study as positive and thought-provoking. Thus, the findings illustrate the applicability of the REIS as an occupation-based environmental assessment, provided that the concept of PCC has been understood by key stakeholders of facilities prior to the assessment, or as part of the cultural change journey. This has implications for occupational therapy practice in the South African context in terms of community and education of aged care sector stakeholders. Occupational therapists need to identify opportunities to act as educators of PCC benefits, not only to residents but also to staff.

### Recommendations for Occupational Therapy Practice in South African RACFs

As the occupational therapy profession struggles to distinguish itself from other, less expensive, therapeutic modalities within RACFs, research needs to focus on evidence-based practices that facilitate collective approaches to PCC. Such an endeavor is supported by the World Federation of Occupational Therapists’ research priorities (Mackenzie et al., 2017). Measurable outcomes and evidence demonstrate professionalism. The REIS presents an opportunity to use metrics in support of suggestions for environmental modifications, as part of an evidence-based approach to practice.

Our findings suggest that occupational therapists practicing in the South African aged care sector need to thoughtfully address organizational culture change, an issue that is perpetually relevant. Thomas (1996) likens culture change to tending a garden. First, the soil needs to be prepared, which includes getting to know and trust the people that you are on the culture change journey with. Thereafter, a seed can be planted (i.e., introducing culture change) and eventually the crop (i.e., successes and improved quality of life for staff and residents) can be harvested. The REIS is one tool that can be used by occupational therapists to facilitate “planting the seed.” However, by first “warming the soil” and promoting an engagement culture within a facility prior to using the REIS, better understanding of its implementation might be achieved. The facilities involved in this study could pioneer practices that can be adopted more widely by RACFs in SA.

### Recommendations for Using the REIS Assessment Tool in Practice

As previously documented by Svensson et al. (2018) and Du Toit et al. (2021), the assessment took longer than the 3 hr per facility, as is the suggested time by the REIS manual. It is recommended that larger facilities are subdivided and assessed systematically to ensure goals for each unit are specific and measurable. Systematic assessments over time can provide a baseline to monitor systematic change.

### Limitations

The first author was employed as an occupational therapist at the time of the study and might have been overly critical during the assessment or, on the contrary, might have not included important information she was acclimatized to. The first author acknowledges a potential power imbalance between herself and the staff and residents she interviewed or observed. This may have caused them to feel exposed or experience distrust toward her and not answer honestly or behave typically.

This study may have limited generalizability as only three aged care facilities participated. However, its findings may have relevance for other facilities with similar organizational and economic environments.

## Conclusion

To ensure longevity of the occupational therapy profession in the South African aged care sector, occupational therapists need to establish a service that adds value to the business and lives of key stakeholders. As illustrated by this study, many RACFs in SA are still embarking on their person-centered culture change journey. There is an opportunity for occupational therapists to utilize the REIS as a tool, alongside management, to build and plan appropriate training or environmental modifications to the benefit of residents and staff. This study adds to the body of knowledge of occupational therapy practice in aged care settings within the Global South, providing therapists with information to strengthen evidence-based clinical practice of how whole environment assessment tools, such as the REIS, could enable meaningful community interventions that promote PCC and culture change.
